# Bilateral nasolabial cysts: a case report

**DOI:** 10.1186/s13256-016-1024-2

**Published:** 2016-09-07

**Authors:** Masaru Sato, Keiichi Morita, Yuji Kabasawa, Hiroyuki Harada

**Affiliations:** 1 Oral and Maxillofacial Surgery, Tsuchiura Kyodo General Hospital, 4-1-1 Otsuno, Tsuchiura, Ibaraki 300-0028 Japan; 2 Division of Oral and Maxillofacial Surgery, Department of Oral Health Sciences, Graduate School of Medical and Dental Sciences, Tokyo Medical and Dental University, 1-5-45 Yushima, Bunkyo-ku, Tokyo, 113-8510 Japan

**Keywords:** Nasoalveolar cysts, Bilateral lesions, Non-odontogenic cysts

## Abstract

**Background:**

Nasoalveolar cysts are rare non-odontogenic cysts that occur beneath the nasal alar region. Few cases of bilateral nasoalveolar cysts have been described.

**Case presentation:**

We report a rare case of a 67-year-old Japanese woman with bilateral nasoalveolar cysts who presented to our department with the chief complaint of a swollen left nasal alar base. Panoramic radiography revealed no abnormalities. Computed tomography and magnetic resonance imaging revealed a well-circumscribed oval lesion at both alar bases. Therefore, bilateral nasoalveolar cysts were clinically diagnosed. Furthermore, these cysts were extirpated under general anesthesia; the aforementioned diagnosis was histopathologically confirmed. No recurrence has been observed 1 year after surgery.

**Conclusions:**

Nasoalveolar cysts are rare. It is necessary to be careful because nasoalveolar cysts can show bilateral occurrence.

## Background

Nasoalveolar cysts are rare non-odontogenic soft tissue cysts that occur inferior to the nasal alar region [1] and account for only 0.7 % of all maxillary and mandibular cysts [2]. Approximately 10 to 11 % of patients with nasoalveolar cysts, which are more frequent in females aged >40 years, show bilateral occurrence [1–11].

Patients with nasoalveolar cysts usually complain of swelling adjacent to their nose. Swelling from the mass can extend into the nasal and oral cavities. Here we describe a case of bilateral nasoalveolar cysts.

## Case presentation

On 24 June 2002, a 67-year-old Japanese woman presented to our department with the chief complaint of a swollen left nasal alar base. She had restrictive lung impairment and was treated for gastric cancer at 60 years. There was no history of any intranasal or intraoral discharge or of oral and maxillofacial trauma, nor were there any signs of perinasal inflammation. There were no noteworthy matters in her social, environmental, and family history.

**Fig. 1:**
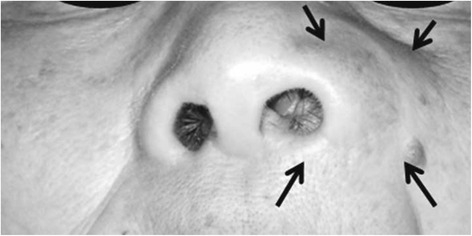
Extra oral findings of the patient at the first visit. A diffuse swelling without tenderness was found at her left nasal alar base (arrows)

An extraoral examination revealed a diffuse swelling of her left nasal alar base without tenderness (Fig. 1). Her left nasolabial fold was less distinct than the right nasolabial fold, and her left ala was deformed because of the markedly elevated alar base.

**Fig. 2:**
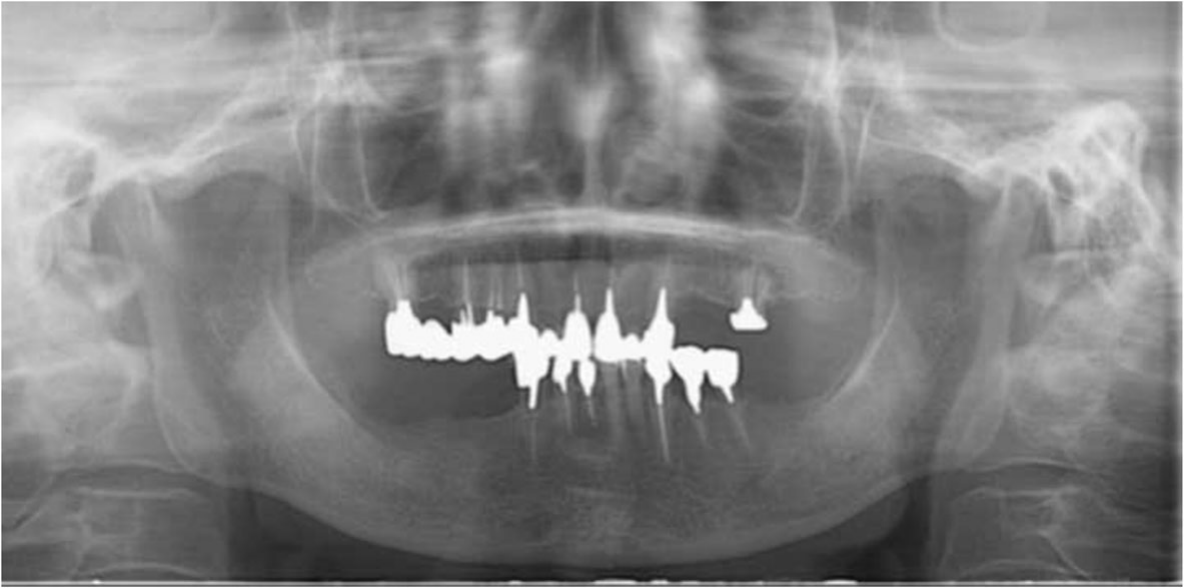
Panoramic radiograph obtained at the first examination. Only apical periodontitis of the left upper central incisor was observed

An intraoral examination revealed a swollen left upper gingivolabial sulcus (Fig. 2). The mucosal surface was normal, and the swelling fluctuated upon palpation but was not tender. No swelling was observed at the right upper gingivolabial sulcus.

**Fig. 3:**
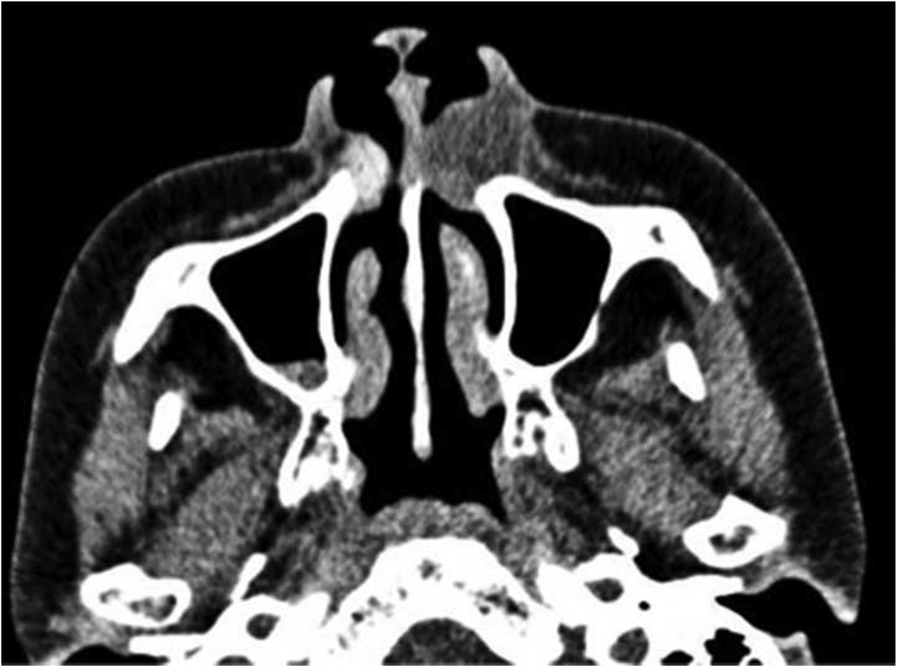
Computed tomography image obtained at the first examination. A well-circumscribed oval lesion, approximately 19 × 14 × 12 mm in size, was found inferior to the left nasal alar base. A small high-density area was observed adjacent to the right piriform aperture

A panoramic radiograph showed no abnormal findings (Fig. 2); however, apical periodontitis of her left upper central incisor was observed. Computed tomography (CT) findings revealed a well-circumscribed oval lesion, approximately 19×14×12 mm in size, inferior to her left nasal alar base (Fig. 3). The lesion was relatively homogeneous and showed a lower density than the muscle. Absorption of the maxilla and nasal septum was not clearly identifiable on the CT images. Although an area of high density was observed adjacent to the right piriform aperture, it was difficult to confirm the presence of a lesion.

**Fig. 4:**
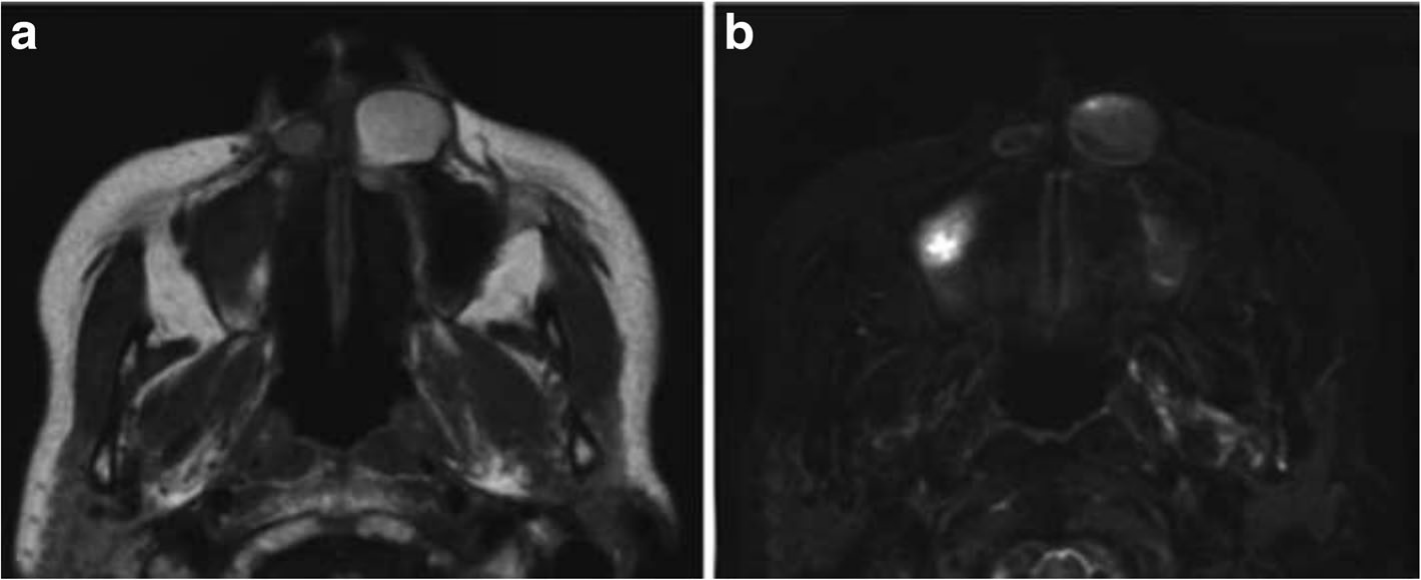
Magnetic resonance images obtained just before surgery. **a**. T1-weighted image. Oval lesions with high internal signal intensity inferior to the bilateral alar base. **b**. T2-weighted short inversion time inversion recovery image showing low signal intensity

Because she declined further evaluation and treatment, a wait-and-see approach was adopted. Although an increase in the swelling was noted in 2005, it remained untreated. Because of the recurrence and further aggravation of the swelling, she finally requested surgical removal of the lesion on 19 January 2010. On 7 May 2010, magnetic resonance imaging (MRI) revealed a cyst-like lesion, measuring approximately 25 mm in maximum diameter, at her left nasal alar base and another cyst-like lesion, measuring approximately 12 mm in maximum diameter, at her right nasal alar base. Both lesions showed high signal intensity on T1-weighted images and low signal intensity on T2-weighted short inversion time inversion recovery images (Fig. 4a, b). On the basis of a clinical diagnosis of bilateral nasoalveolar cysts, the cysts were extirpated under general anesthesia on 19 May 2010. The cysts adhered relatively strongly to the mucosa of her nasal cavity floor and the levator labii superioris alaeque nasi muscle. No maxillary reabsorption was observed on either side. Her postoperative course was uneventful, and no recurrence was observed 1 year after surgery.

**Fig. 5:**
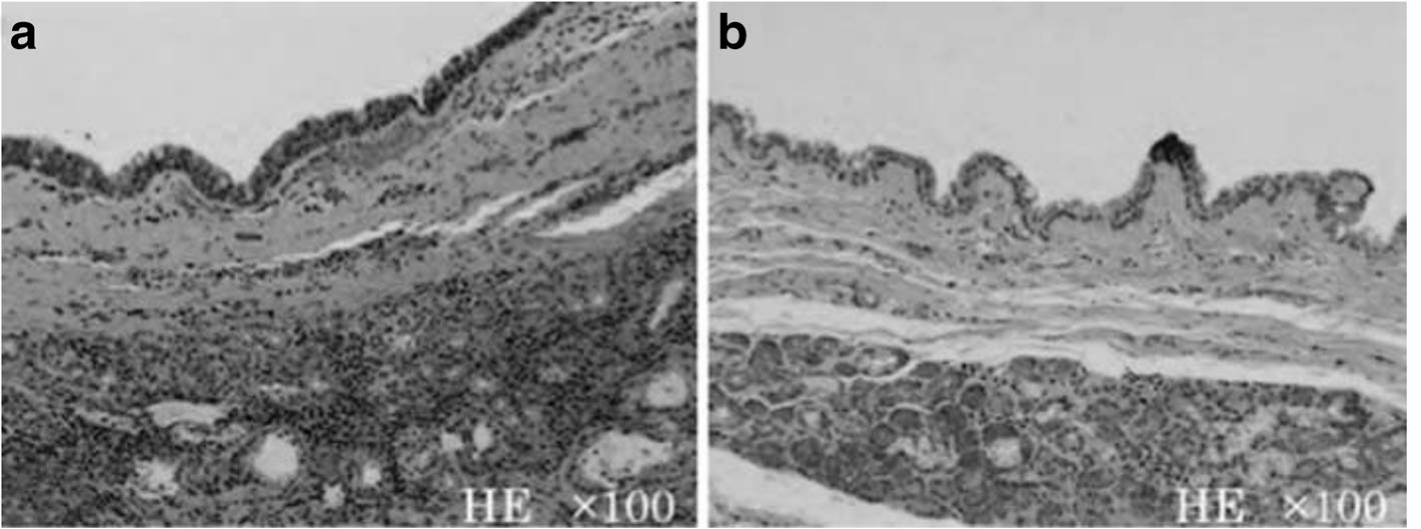
Hematoxylin and eosin-stained sections of the surgical specimen. **a**. Right-sided lesion. **b**. Left-sided lesion. The cysts were lined with thin, non-keratinized squamous epithelium containing a number of goblet cells. The cyst walls comprised fibrous connective tissue with seromucous glands

A histopathological examination showed that both cysts were lined with single-layered or multi-layered non-keratinized squamous epithelia, which comprised several goblet cells. The cyst walls were composed of a fibrous connective tissue with mild inflammatory cell infiltration and contained seromucous glands in the deeper layers (Fig. 5a, b). The histopathological diagnosis was bilateral nasoalveolar cysts.

## Discussion

Nasoalveolar cysts are non-odontogenic cysts that occur in the nasal alar region. They were first described in 1882 [3], and a theory of their origin was proposed by Klestadt [6], who suggested that the cysts derive from the epithelium trapped in the line of fusion between the lateral nasal and maxillary processes [12]. However, lack of evidence supporting the idea of the aforementioned entrapment in this location prompted many researchers to discard this hypothesis [2]. Nasoalveolar cysts are presently assumed to develop from triggering events such as trauma or infection. These events may stimulate the otherwise dormant epithelial tissue to develop into a cystic structure [1].

Nasoalveolar cysts account only for 0.7 % of all maxillary and mandibular cysts, occurring approximately in 1 out of 20,000 patients [3]. Unilateral nasoalveolar cysts occur equally on the right or left side of the nose [13].

Roed-Petersen reported that among 116 patients with nasoalveolar cysts, 13 showed bilateral cysts [9]. Vasconcelos *et al*. reported that bilateral occurrence accounts for 6.6 % cases of cysts [13]. Choi *et al*. reported that no bilateral nasoalveolar cysts were observed among 18 patients [2]. Bilateral nasoalveolar cysts appear to be very rare. We treated 20 patients with nasoalveolar cysts between 1965 and 2014, including only one case with bilateral presentation. Table 1 summarizes related reports published since 1967.

**Table 1:** Summary of clinical cases of nasoalveolar cysts published since 1967

References	Number of cases	Sex	Number of bilateral cases
Choi *et al*., 2002 [2]	18	5 M; 13 F	0
Yuen *et al*., 2007 [14]	17	6 M; 11 F	1
el-Din and el-Hamd, 1999 [3]	8	1 M; 7 F	1
Kuriloff, 1987 [7]	26	7 M; 19 F	1
Roed-Petersen, 1969 [9]	116	26 M; 90 F	13
Vasconcelos *et al*., 1999 [13]	15	2 M; 13 F	1
Kato *et al*., 2007 [5]	3	1 M; 2 F	0
Walsh-Waring, 1967 [10]	6	1 M; 5 F	0
Our department	20	2 M; 18 F	1

*F* female, *M* male

It has been reported that nasoalveolar cysts have a significant predilection for women (with a female-to-male ratio of 3.5:1 and 6.5:1) [9, 13]. These cysts most commonly occur in adults, with a maximum prevalence in the fourth and fifth decades of life [12]. Although the overall incidence of nasoalveolar cysts in the Western world is relatively low [11], it has been suggested that this condition occurs more frequently in African Americans [8].

The clinical features are fairly typical among cases. Patients usually complain of a swelling adjacent to the nose [12]. The cyst is asymptomatic unless nasal obstruction occurs or it is infected. The infection can spread locally and cause tenderness, swelling of the lip and cheek, and facial deformity [12]. In the present case, although the patient visited our hospital immediately after she first noted the swelling, the cysts may have existed for a long period of time before presentation. Because of the absence of recognizable pain and on her request, 8 years passed before the surgery was performed. In her case, the presence of apical periodontitis of her left upper central incisor indicated that the infection may have spread locally.

Panoramic and intraoral radiographs usually reveal no abnormalities in cases of nasoalveolar cysts [5]. Therefore, the best technique to help in the diagnosis of a nasoalveolar cyst is the aspiration of the cyst fluid and replacement of the fluid with a contrast medium to visualize the lesion on radiographs; however, the risk of infection should be weighed against the advantages of this procedure [13]. CT and MRI accurately demonstrate nasoalveolar cysts; thus, these techniques are useful for diagnosis and surgical treatment planning [5]. However, because the viscosity of the cyst fluid varies [5], CT may not clearly reveal the density of the fluid. On the other hand, on MRI, the cysts often appear as low signal intensity (equal to that of muscle) masses on T1-weighted images and high signal intensity masses on T2-weighted images [5]. Therefore, MRI is considered to be more useful for image diagnosis than CT. Our patient noted a swollen left nasal alar base, and a cystic lesion was confirmed using CT images. Her initial CT images also showed small heterogeneous opacity on the right; however, we were unable to confirm the presence of a lesion at that time because she declined further examinations. MRI performed 8 years later revealed bilateral nasoalveolar cysts. Because nasoalveolar cysts occur within the soft tissue and their contents and properties are variable among cases, MRI is an indispensable tool for presurgical diagnosis.

In our case, an area of high density was also observed adjacent to her right piriform aperture, but the density was lower than that of her left piriform aperture. Considering the fact that unenhanced CT showed low-to-high density cysts in our case, we believe that nasoalveolar cysts at the piriform aperture may be particularly difficult to diagnose.

On histopathological examination, nasoalveolar cysts are often lined by stratified squamous, cuboidal, or respiratory epithelium [12]. The cyst wall lining comprises fibrous connective tissue abundant in collagen fibers, with numerous inflammatory cells in the subepithelial region [12], and goblet cells are often observed [2, 3, 9, 11, 13]. Roed-Petersen reported that goblet cells were found in 67 % of patients [9], whereas Choi *et al*. reported a frequency of only 55.6 % [2]. In our case, both cysts showed mild infiltration of inflammatory cells in the connective tissue area; the epithelial lining contained several goblet cells, and the deeper layers of the cyst walls were rich in seromucous glands.

Treatment for nasoalveolar cysts is generally the complete extirpation of the cyst through an intraoral approach via the gingivolabial sulcus on the affected side. In recent years, endoscopic surgery has also been reported [14]. Recurrence is very rare [2], and malignant transformation is an unusual phenomenon, which has been documented in only one case [15]. Therefore, careful histopathological examinations and long-term postoperative follow-ups are recommended for nasoalveolar cysts, although they are nonaggressive and rarely occur as recurrent benign lesions.

Because a nasoalveolar cyst is a soft tissue mass, MRI is most helpful for its differential diagnosis with respect to the nasopalatine duct and globulomaxillary cysts, although both these entities are intraosseous. Other differential diagnoses include epidermoid or dermoid cysts, unilocular lymphatic malformations, postoperative maxillary cysts, odontogenic cysts, periapical dental abscesses, and furunculosis. On clinical examination, soft tissue masses, including benign tumors (neurogenic tumor or hemangioma) or malignant tumors (squamous cell carcinoma or minor salivary gland tumor), should also be considered. A diagnosis of nasoalveolar cysts should be considered when unilateral or bilateral lesions are present in the nasal alar region and CT and MRI demonstrate characteristic location and features.

## Conclusions

We treated a case of nasoalveolar cyst. Nasoalveolar cysts are rare. It is necessary to be careful because nasoalveolar cysts can show bilateral occurrence.

## Abbreviations

CT: Computed tomography
MRI: Magnetic resonance imaging
